# A prospective study on phototherapy induced hypocalcemia in neonates with hyperbilirubinemia

**DOI:** 10.6026/973206300223510

**Published:** 2026-06-30

**Authors:** Sreekanth Kanumilli, Geshmanjali Kakarala, Lalita Wadhwa

**Affiliations:** 1 Department of Paediatrics, GITAM Institute of Medical Sciences and Research, GITAM Deemed to be University, Rushikonda, Visakhapatnam, Andhra Pradesh, India

**Keywords:** Calcium, bilirubin, phototherapy, hypocalcemia, neonate, jaundice

## Abstract

Phototherapy is used to treat neonatal hyperbilirubinemia and leads to hypocalcemia in newborns and how frequently and severely this complication occurs. Therefore, it is of interest to assess the incidence of hypocalcemia after 48 hours of phototherapy and compare its severity between term and pre-term newborns. Blood samples were collected and analyzed for total and direct bilirubin by diazo method and calcium by Arsenazo III method and the phototherapy was given to neonates who required it and followed according to AAP guidelines. The mean total bilirubin decreased from 13.76 ± 1.63 mg/dl before phototherapy to 6.87 ± 0.57 mg/dl after phototherapy and 2% of neonates developed hypocalcemia (<7 mg/dl). Hypocalcemia is less common and is seen in term neonates receiving phototherapy with calcium supplementation.

## Background:

Newborn hyperbilirubinemia is the most commonly detected abnormal physical finding, with more than two-thirds of babies experiencing severe jaundice [1]. For most neonates, unconjugated hyperbilirubinemia is a typical physiological occurrence. During the first week of life, 60% of term and over 80% of pre-term newborns suffer from neonatal hyperbilirubinemia [2]. About 6.1% of full-term newborns have bilirubin levels above 12.9 mg/dL and untreated severe unconjugated hyperbilirubinemia can lead to neurotoxicity [3]. Neonatal hyperbilirubinemia is a sign of an underdeveloped bilirubin excretion pathway of the liver. Early postnatal discharge from hospitals is one of the reasons for readmission of newborns again within their first few days of life [4]. Compared to term neonates, premature newborns experience neonatal jaundice at a significantly higher frequency, which calls for therapeutic intervention [5]. Increased amounts of unconjugated bilirubin can cause serious, lifelong problems like bilirubin encephalopathy and kernicterus [6]. Conjugated hyperbilirubinemia is indicative of impending systemic infections or severe liver damage [7]. Treatment options for hyperbilirubinemia include phototherapy, exchange transfusions and pharmaceuticals [8]. Bilirubin is changed by phototherapy into isomers that are soluble in water and can be excreted without going through the liver's conjugation process [9]. Phototherapy effectiveness depends on factors like light type, distance, exposed area, cause of jaundice and initial TSB level [10]. Phototherapy can have unfavorable side effects, including dehydration, heat exhaustion, genotoxicity, loose stools, skin rashes and hypocalcemia [11]. Several studies shown to check the safety of phototherapy in the treatment of newborn hyperbilirubinemia. Compared to other side effects, there are currently very few studies showing how phototherapy negatively affects serum electrolytes. One of the known side effects is hypocalcemia [12]. Following phototherapy, 75% of full-term and 90% of preterm babies experienced hypocalcemia. Irritability, jitteriness, convulsions and hypocalcemia can lead to serious consequences like apnea [13, 14, 15]. Therefore, it is of interest to treat the neonatal hyperbilirubinemia and leads to hypocalcemia in newborns and how frequently and severely this complication occurs.

## Materials:

## Source of data:

Inborn Neonates admitted to NICU with hyperbilirubinemia who met the inclusion and exclusion criteria were included in the study.

## Type of study:

The present study is a hospital-based prospective observational study

## Inclusion criteria:

Term and late Pre-term AGA neonates with unconjugated hyperbilirubinemia requiring phototherapy according to AAP guidelines

## Exclusion criteria:

Newborns with jaundice in the initial 24 hours of life; icterus lasting more than 14 days; born to a diabetic mother; neonatal sepsis; birth asphyxia; babies who had exchange transfusion or were on TPN.

## Sample size:

Postulating the probability of type 1 error as 0.05 with a power of 0.8, paired difference to be recognized as 0.2 and expected Standard Deviation from the previous study as 0.8, the required sample size of 50 was calculated, who met the inclusion and exclusion criteria were included in the study.

## Patient informed consent:

Written consent was taken from the parents.

## Institutional ethics committee clearance: 

The study was conducted after taking the ethical clearance from the institutional ethical committee.

## Methodology: 

Sepsis screening is done wherever required. Blood samples were collected with aseptic precautions drew the blood samples and no squeezed samples were used and the samples were analyzed immediately within 15 minutes after drawing blood. Total and direct bilirubin measured by the diazo method (Diazotized sulfanilic test). In this test, bilirubin interacts with diazotized sulfanilic acid in the presence of ethylene glycol and dimethyl sulfoxide to produce an intensely colored diazo dye, i.e., azobilirubin. The intensity of the colored diazo dye is proportional to the quantity of bilirubin. Measurement of calcium was done by Arsenazo III method. Arsenazo III combines with calcium ions at pH6.5 to form colored chromophores; the absorbance of the chromophore is measured at 650 nm and is proportional to calcium concentration. Both calcium and bilirubin measured by an automated analyzer COBAS311. The sample was analyzed immediately within 15 minutes after drawing blood. Phototherapy was given by blue fluorescents lamps (410-470nm) placed at 30 to 40 cms distance from the neonates who required phototherapy according to AAP guidelines. The eyes and genitalia were covered with the head exposed to phototherapy. Gestational age of 37 completed to < than 41weeks 6 days (i.e., 259 to 293days). Neonatal Period: It refers to a period from birth to 28 days of postnatal life.

## Hypocalcemia:

Serum calcium level of less than 7.0mg/dl is defined as hypocalcemia.

## Statistical analysis: 

Neonatal and Maternal data were collected in predesigned proforma. Data shall be analyzed using SPSS 22.0 and R environment ver.3.2.2 was used for the analysis and evaluation of the data and Microsoft Word and MS Excel have been used to generate graphs, tables, etc. For quantitative data, mean and standard deviation (SD) were calculated for qualitative data percentages calculated. A chi-square test was used for comparing differences between categorical variables. For comparison between the means, Wilcoxson matched test was used and the students t-test was used. For interpretation of results, significance shall be adopted at p-value < 0.05 at a 95% confidence interval.

## Results:

In current study, 27(54%) of neonates were males whereas 23(46%) of neonates were females with male predominance. It was also found that, 52% of neonates belong to 60 to 90hours, 39.3% of neonates belong to more than 90hours and 8.7% of neonates belong to less than 60hours with mean hours of life of 83.52 ± 15.18 hours (Table 1) (Figure 1 - see PDF). The results showed that 60% of neonates had weight of less than 3kg whereas 36% of neonates had weight of 3kg to 3.5kg and 4% of neonates had weight of more than 3.5kg. The mean weight of the neonates was 3.05 ± 0.28kgs (Table 2). 74% of neonates are born of gestational age of 38 to 39 weeks whereas 26% of neonates are born of gestational age of 36 to 37 weeks (Table 3). In current study we also found that 37(74%) of neonates were term babies and 13(26%) of neonates were pre-term babies. 32(64%) of neonates were delivered by LSCS whereas 18(36%) of neonates were delivered by normal vaginal delivery. In current study, the mean total bilirubin before phototherapy was 13.76 ± 1.63mg/dl whereas the mean total bilirubin after phototherapy was 6.87 ± 0.57mg/dl where there was statistically significant decrease in total bilirubin after phototherapy (P value <0.0001) (Table 4). The study found that, the mean serum calcium before phototherapy was 9.65 ± 0.64mg/dl whereas the mean serum calcium after phototherapy was 8.76 ± 0.62mg/dl where there was statistically significant decrease in serum calcium after phototherapy (P value <0.0001) (Table 5). 47(94%) of neonates had decreased serum calcium levels whereas 3(6%) of neonates had same serum calcium levels. Figure 1 (see PDF) depicted the distribution of neonates based on the levels of serum bilirubin and calcium levels. From the study findings, it was found that 1(2%) of neonate had hypocalcemia (<7mg/dl) after phototherapy whereas 49(98%) of neonates had normal serum calcium levels. In current study, among the male neonates, the mean serum calcium before phototherapy was 9.7 ± 0.59mg/dl whereas after phototherapy was 8.8 ± 0.62mg/dl where there was significantly decrease in serum calcium after phototherapy (P value <0.001). Among female neonates, the mean serum calcium before phototherapy was 9.58 ± 0.69mg/dl whereas after phototherapy was 8.72 ± 0.63mg/dl where there was significantly decrease in serum calcium after phototherapy (P value 0.000) (Table 6). Among the term neonates, the mean serum calcium before phototherapy was 9.84 ± 0.57mg/dl whereas after phototherapy was 8.96 ± 0.58mg/dl where there was significantly decrease in serum calcium after phototherapy (P value <0.001). Among pre-term neonates, the mean serum calcium before phototherapy was 9.02 ± 0.39mg/dl whereas after phototherapy was 8.13 ± 0.15mg/dl where there was significantly decrease in serum calcium after phototherapy (P value < 0.001) (Table 7). Among neonates born by normal vaginal delivery, the mean calcium before phototherapy was 10.04 ± 0.44mg/dl whereas after phototherapy was 9.01 ± 0.65mg/dl where there was significant decrease in serum calcium (P value <0.001) after phototherapy. Among neonates born by LSCS, the mean calcium before phototherapy was 9.43 ± 0.62mg/dl whereas after phototherapy was 8.63 ± 0.56mg/dl where there was significant decrease in serum calcium after phototherapy (P value <0.001) (Table 8). In current study, the mean serum calcium among neonates with weight less than 3kg before phototherapy was 9.47 ± 0.59mg/dl whereas after phototherapy was 8.57 ± 0.58mg/dl where there was significant decrease in serum calcium after phototherapy (P value <0.001). Among neonates with more than 3.1kg, the mean serum calcium before phototherapy was 9.91 ± 0.61mg/dl whereas after phototherapy was 9.06 ± 0.57mg/dl where there was significant decrease in serum calcium after phototherapy (P value 0.000) (Table 9) (Figure 2 - see PDF).

## Discussion:

A major issue during the infant stage is jaundice. Even in term neonates, elevated bilirubin levels have the potential to harm the growing central nervous system and result in permanent neurological damage. In the first week of life, about 60% of term babies and 80% of preterm babies exhibit icteric symptoms. It is typically benign and doesn't need to be treated in most cases. Five to ten percent of them have hyperbilirubinemia that is clinically severe, necessitating the use of phototherapy [16]. The liver's immature bilirubin excretory system is the primary cause of newborn hyperbilirubinemia. In the current era of postnatal discharge from the hospital, it is the most frequent cause of re-admission for neonates within their first week of life [17]. Both pediatricians and parents should be concerned when their newborn exhibits jaundice [18]. High amounts of unconjugated non-hemolytic bilirubin can cause kernicterus, a condition that results in severe, lifelong neurodevelopmental impairments and bilirubin encephalopathy [19]. Conjugated hyperbilirubinemia is a sign of systemic illnesses or potentially serious liver abnormalities. Therefore, it is crucial to properly diagnose and treat neonatal hyperbilirubinemia. The three main forms of treatment are phototherapy, exchange transfusion and medication. To treat neonatal hyperbilirubinemia, phototherapy is crucial. Unwanted side effects from phototherapy treatments include skin rashes, diarrhea, retinal degenerations, fever, nasal obstruction, bronze baby syndrome and others [20]. However, the hypothesis posits that phototherapy impedes the pineal secretion of melatonin, hence impeding the impact of cortisol on calcium levels in bone. Uncontrolled cortisol enhances calcium absorption by the bone and causes a direct hypercalcemic spike [21]. Numerous biological functions, including blood coagulation, cell membrane integrity and function, neuromuscular excitability and cellular enzymatic activity, depend on calcium. Cell membrane excitability and permeability to sodium ions are both enhanced by hypocalcemia. Apnea, seizures, jitters, irritability, increased extensor tone, hyperreflexia and stridor (laryngospasm) are examples of non-specific symptoms [22, 23, 24, 25]. In current study, 54% of neonates were males whereas 46% of neonates were females with male predominance. In a study done by Alshaymaa *et al.* [26] showed that 52.5% of patients were males and 47.5% of patients were females. Nazim *et al.* [27] showed that 52% of neonates were males whereas 48% of neonates were females. Tosson *et al.* [28] showed that 54.6% of neonates were males whereas 45.4% of neonates were females. Khalil *et al.* [29] showed that 56% of cases were males whereas 44% of cases were females. In current study, 60% of neonates had weight of less than 3kg whereas 36% of neonates had weight of 3kg to 3.5kg and 4% of neonates had weight of more than 3.5kg. The mean weight of the neonates was 3.05 ± 0.28kgs and the results were similar to the reports of Tosson *et al.* and Khalil *et al.* [28, 29]. In current study, 64% of neonates were delivered by LSCS whereas 36% of neonates were delivered by normal vaginal delivery. In a study done by Reddy and Krishnappa [30] showed that 44.3% of patients were delivered by normal vaginal delivery whereas Neu and Rushing [31] showed that 42.2% of neonates were delivered by normal vaginal delivery whereas 57.8% of neonates were delivered by LSCS. In current study, the mean total bilirubin before phototherapy was 13.76 ± 1.63mg/dl whereas the mean total bilirubin after phototherapy was 6.87 ± 0.57mg/dl. Alshaymaa *et al.* [26] showed that the mean total bilirubin before phototherapy was 17.20 ± 1.87mg/dl whereas the mean total bilirubin after phototherapy was 11.91 ± 1.64mg/dl whereas Nazim *et al.* [27] showed that that the mean total bilirubin before phototherapy was 20.83 ± 9.29mg/dl whereas the mean total bilirubin after phototherapy was 12.91 ± 5.11mg/dl. Akpinar *et al.* [32] showed that the mean total bilirubin before phototherapy was 18.4mg/dl whereas the mean total bilirubin after phototherapy was 10.5mg/dl where there was statistically significant decrease in total bilirubin after phototherapy (p-value <0.0001). In current study, the mean serum calcium before phototherapy was 9.65 ± 0.64mg/dl whereas the mean serum calcium after phototherapy was 8.76 ± 0.62mg/dl where there was statistically significant decrease in serum calcium after phototherapy (p-value <0.0001). 94% of neonates had decreases serum calcium levels whereas 6% of neonates had same serum calcium levels. In a study done by Alshaymaa *et al.* [26] showed that the mean serum calcium before phototherapy was 9.22 ± 0.99mg/dl whereas the mean serum calcium after phototherapy was 8.60 ± 1.21mg/dl where there was statistically significant decrease in serum calcium after phototherapy (p-value 0.002). The findings of the present study were in accordance with the findings of Nazim *et al.* [27] and Shahriarpanah *et al.* [33] showed that the mean serum calcium before phototherapy was 8.90 ± 0.55mg/dl whereas the mean serum calcium after phototherapy was 8.33 ± 0.72mg/dl. In a study done by Vigneshwar *et al.* [34] showed that the mean serum calcium before phototherapy was 8.91 ± 0.4mg/dl whereas the mean serum calcium after phototherapy was 8.34 ± 0.28mg/dl. In current study, 2% of neonates had hypocalcemia (<7mg/dl) after phototherapy whereas 98% of neonates had normal serum calcium levels. Neu and Rushing [31] reported 27% of cases had hypocalcemia after phototherapy Akpinar *et al.* [32] showed that 23.3% of cases had hypocalcemia after phototherapy whereas. Khan *et al.* [35] and Ravindranath *et al.* [36] revealed that 23.68% and 28% of neonates had hypocalcemia after phototherapy whereas Pereira *et al.* and Manazir Hasan *et al.* concluded that there is significant decrease in the levels of calcium after phototherapy [37, 38].

## Conclusion:

Data shows that decrease in serum calcium is significant after phototherapy, a frequently employed therapeutic approach for neonatal hyperbilirubinemia. Even though hypocalcemia is less common, it is in that term neonates receiving phototherapy and there is significant decrease in serum calcium levels. This means that serum calcium levels should be monitored for at least 48 hours in these neonates and calcium supplementation needs to be taken into consideration.

## Advancement to knowledge:

This study provides prospective evidence regarding the frequency and clinical significance of phototherapy-induced hypocalcemia in neonates with hyperbilirubinemia and highlights the importance of monitoring serum calcium during phototherapy to improve neonatal safety and outcomes.

## Figures and Tables

**Table 1:** Distribution of neonates according to hours of life

**Hours of life**	**Number of neonates**	**%**
<60	4	8.7
60-90	26	52
>90	20	39.3
Total	50	100
Mean ± SD	83.52 ± 15.18hrs	
Range	54-112hrs	

**Table 2:** Distribution of neonates according to weight

**Weight (kgs)**	**N**	**%**
<3.0	30	60
3.0 - 3.5	18	36
>3.5	2	4
Total	50	100
Mean ± SD	3.05 ± 0.28kg	
Range	2.55 - 3.66kg	

**Table 3:** Distribution of neonates according to gestational age

**Gestational age (weeks)**	**Number of neonates**	**%**
36 - 37	13	26	
38 - 39	37	74	
Total	50	100	
Mean ± SD	37.78 ± 1.11weeks	
Median	38weeks	

**Table 4:** Distribution of neonates according to mean serum total bilirubin

**Serum Total bilirubin (mg/dl)**	**Mean**	**SD**	**P-value**
Before phototherapy13.76	1.63	<0.0001	
After phototherapy6.87	0.57		

**Table 5:** Distribution of neonates according to mean serum calcium

**Serum calcium (mg/dl)**	**Mean**	**SD**	**P value**
Before phototherapy	9.65	0.64	<0.0001
After phototherapy	8.76	0.62	

**Table 6:** Comparison of Gender vs Serum calcium among neonates

**Gender**	**Phototherapy**	**Serum calcium (mg/dl)**		**P value**
		**Mean**	**SD**	
Male	Before	9.7	0.59	<0.001
	After	8.8	0.62	
Female	Before	9.58	0.69	0
	After	8.72	0.63	

**Table 7:** Distribution of neonates according to term/pre-term neonates and serum calcium

**Term/preterm**	**Phototherapy**	**Serum calcium (mg/dl)**		**P value**
		**Mean**	**SD**	
Term	Before	9.84	0.57	<0.001
	After	8.96	0.58	
Pre-term	Before	9.02	0.39	<0.001
	After	8.13	0.15	

**Table 8:** Distribution of neonates according to mode of delivery and serum calcium

**MOD**	**Phototherapy**	**Serum calcium (mg/dl)**		**P value**
		**Mean**	**SD**	
NVD	Before	10.04	0.44	<0.001
	After	9.01	0.65	

**Table 9:** Distribution of neonates according to their weight and serum calcium

**Weight (kg)**	**Phototherapy**	**Serum calcium (mg/dl)**		**P value**
		**Mean**	**SD**	
≤ 3.0	Before	9.47	0.59	<0.001
	After	8.57	0.58	
≥ 3.1	Before	9.91	0.61	0
	After	9.06	0.57	
